# Long-Term Exposure to Low-Level Arsenic in Drinking Water and Diabetes Incidence: A Prospective Study of the Diet, Cancer and Health Cohort

**DOI:** 10.1289/ehp.1408198

**Published:** 2014-06-13

**Authors:** Elvira Vaclavik Bräuner, Rikke Baastrup Nordsborg, Zorana Jovanovic Andersen, Anne Tjønneland, Steffen Loft, Ole Raaschou-Nielsen

**Affiliations:** 1 Diet, Genes and Environment, Danish Cancer Society Research Centre, Copenhagen, Denmark; 2 Danish Building Research Institute, Aalborg University, Aalborg, Denmark; 3 Center for Epidemiology and Screening, and; 4 Section of Environmental Health, Department of Public Health, Faculty of Health Sciences, University of Copenhagen, Copenhagen, Denmark

## Abstract

Background: Established causes of diabetes do not fully explain the present epidemic. High-level arsenic exposure has been implicated in diabetes risk, but the effect of low-level arsenic exposure in drinking water remains unclear.

Objective: We sought to determine whether long-term exposure to low-level arsenic in drinking water in Denmark is associated with an increased risk of diabetes using a large prospective cohort.

Methods: During 1993–1997, we recruited 57,053 persons. We followed each cohort member for diabetes occurrence from enrollment until 31 December 2006. We traced and geocoded residential addresses of the cohort members and used a geographic information system to link addresses with water-supply areas. We estimated individual exposure to arsenic using all addresses from 1 January 1971 until the censoring date. Cox proportional hazards models were used to model the association between arsenic exposure and diabetes incidence, separately for two definitions of diabetes: all cases and a more strict definition in which cases of diabetes based solely on blood glucose results were excluded.

Results: Over a mean follow-up period of 9.7 years for 52,931 eligible participants, there were a total of 4,304 (8.1%) diabetes cases, and 3,035 (5.8%) cases of diabetes based on the more strict definition. The adjusted incidence rate ratios (IRRs) per 1-μg/L increment in arsenic levels in drinking water were as follows: IRR = 1.03 (95% CI: 1.01, 1.06) and IRR = 1.02 (95% CI: 0.99, 1.05) for all and strict diabetes cases, respectively.

Conclusions: Long-term exposure to low-level arsenic in drinking water may contribute to the development of diabetes.

Citation: Bräuner EV, Nordsborg RB, Andersen ZJ, Tjønneland A, Loft S, Raaschou-Nielsen O. 2014. Long-term exposure to low-level arsenic in drinking water and diabetes incidence: a prospective study of the Diet, Cancer and Health cohort. Environ Health Perspect 122:1059–1065; http://dx.doi.org/10.1289/ehp.1408198

## Introduction

The prevalence and incidence of diabetes is rapidly increasing in all countries, including Denmark, presenting a major public health threat [Carstensen et al. 2008; Danaei et al. 2011; World Health Organization (WHO) 2011]. Established risk factors are mainly related to lifestyle and include older populations, obesity, and physical inactivity and are in part related to a family history of diabetes and genetic polymorphisms. However, these factors do not fully explain the present diabetes epidemic. Given that almost 400 million persons had diagnosed diabetes worldwide in 2008 (Danaei et al. 2011; WHO 2011) and the severe, long-term consequences of this disease in terms of morbidity, mortality, and economic costs, there is an increased need to understand the effects of nontraditional risk factors such as environmental chemicals.

Arsenic occurs in both organic and inorganic environmental forms (Eyre et al. 2004; Mandal and Suzuki 2002). Organic arsenic is found primarily in food, whereas inorganic arsenic is mostly found in aquifers (Eyre et al. 2004; Mandal and Suzuki 2002) where it accumulates by natural processes such as weathering and erosion (Smedley 2008). Globally, exposure to inorganic arsenic via groundwater used for drinking is associated with most health risks (Smedley and Kinniburg 2005). In Denmark, all drinking water from tap water is derived from groundwater (Dansk Vand og Spildevandsforening 2010); this tap water is very clean and not chlorinated and is bottled-water quality at the tap (Thomsen et al. 2004). It is the standard in Denmark to use tap water for cooking, coffee, tea, and drinking. Thus, the consequences of a possible relationship between low-level groundwater arsenic exposure and population health are serious.

Arsenic exposure has been implicated in the diabetes epidemic. Mechanisms remain unclear, but based on *in vitro* studies, they are thought to include the disruption of several pathways related to pancreatic β-cell function and insulin sensitivity, including oxidative stress, glucose uptake and transport, gluconeogenesis, adipocyte differentiation, and calcium ion signaling (Díaz-Villaseñor et al. 2007; Druwe and Vaillancourt 2010; Tseng 2004). Two recent systematic reviews and a meta-analysis of epidemiological studies addressing the association between arsenic exposure in drinking water and diabetes risk have concluded that the positive association of diabetes with high-level inorganic arsenic exposure was consistent but also that the evidence regarding low-level exposure, defined as < 50 ppb (equivalent to 50 μg/L), remains unclear and that a threshold might exist (Maull et al. 2012; Navas-Acien et al. 2006; Wang et al. 2013). The role of low-level arsenic in diabetes risk needs to be elucidated, and the need for future research including large prospective studies in areas of low arsenic exposure using individual arsenic exposures has been recommended (Maull et al. 2012).

The Danish Diet, Cancer and Health (DCH) cohort is a large prospective study, with detailed information on potential confounders collected at baseline, and the Danish National Diabetes Register (NDR) (Carstensen et al. 2008, 2011) allows for the objective ascertainment of diabetes on a national scale. By combining geocoded past and present residential addresses of cohort participants—obtained from the Danish Civil Registration System (CRS) (Pedersen 2011)—with geographic information on water supply areas, the estimation of individual arsenic exposure of all cohort participants was made possible.

The purpose of this large population-based prospective study was to determine whether individual long-term exposure to low-level inorganic arsenic in drinking water is associated with an increased risk of diabetes.

## Methods

*The DCH cohort*. The present prospective cohort study was based on the prospective DCH cohort comprising 57,053 participants, 50–64 years of age, enrolled in 1993–1997 (Tjønneland et al. 2007). To be eligible for enrollment, the participants had to have been born in Denmark, be living in the Copenhagen or Aarhus areas at the time of enrollment, and not have a cancer diagnosis registered in the Danish Cancer Registry at the time of enrollment (Tjønneland et al. 2007). The baseline examination included a self-administered, interviewer-checked, questionnaire on diet, beverages, present and previous smoking habits, length of school attendance, and other items related to health, lifestyle, and socioeconomic status (SES) (Overvad et al. 1991; Tjønneland et al. 1991, 2007). Height, weight, and waist circumference were measured by trained staff according to standardized protocols. The present study was approved by the Scientific Ethics Committee for Copenhagen and Frederiksberg and The Danish Data Protection Agency, and written informed consent was obtained from all participants before enrollment.

Since the establishment of the CRS (Pedersen 2011) in 1968, all citizens of Denmark are assigned a unique personal identification number, which allows accurate linkage between registers. We used the CRS to obtain information on date of death, emigration or disappearance of the cohort members, and information on past and present residential addresses.

*The Danish NDR*. Using personal identification numbers, we linked the cohort members to the NDR to identify incident diabetes cases between baseline (1993–1997) and either death, emigration, disappearance, or end of follow-up (31 December 2006).

The NDR was established in 2006 by the Danish National Board of Health to describe and monitor the occurrence of diabetes in Denmark and to provide data for epidemiological research. Establishment of this register has been previously discussed in detail (Carstensen et al. 2008, 2011). In brief, the NDR links three existing nationwide administrative records in the Danish health care system: *a*) the National Patient Register (Lynge et al. 2011), containing hospital and outpatient clinic discharge diagnoses since 1994; *b*) the National Health Services Register (Andersen et al. 2011), with information of all services provided by general and specialist practitioners since 1973; and *c*) the National Prescription Registry (Kildemoes et al. 2011), containing all prescriptions dispensed at Danish pharmacies since 1993 (Carstensen et al. 2011).

Inclusion in the NDR required one or more of the following criteria: *a*) a diabetes hospital discharge diagnosis in the National Patient Register (Lynge et al. 2011) defined according to the *International Classification of Diseases* (ICD), *10th Revision*, ICD-10 codes E10–14, H36.0, and O24 (excluding DO24.4), or the *8th Revision*, ICD-8 codes 249 and 250; *b*) a record of chiropody for diabetic patients in the National Health Services Register (Andersen et al. 2011); *c*) a record of five or more blood glucose measurements within 1 year or two blood glucose measurements per year in 5 consecutive years in the National Health Service Register (Andersen et al. 2011); and *d*) a record of a second purchase of insulin or of oral glucose-lowering drugs within 6 months in the National Prescription Registry (Kildemoes et al. 2011). At least two inclusion criteria were met by 60% of all patients in the NDR and 47% met at least three criteria (Carstensen et al. 2008). Because of the different dates of initiation of the underlying registers and the accumulation of prevalent cases, only incidence values after 1 January 1995 were found to be reliable (Carstensen et al. 2008). Thus, we defined the incidence of diabetes as the earliest record in the diabetes register occurring after 1 January 1995, between baseline (1993–1997) and 31 December 2006. In addition to this original NDR definition (all diabetes), we also defined a more strict definition of incidence (“strict diabetes”) by excluding persons who were included in the NDR solely as a result of a blood glucose test because a number of these people may not have actually had diabetes. We have previously used this register and these two definitions to assess associations between exposure to air pollution and the risk of developing diabetes (Andersen et al. 2012).

*Residential histories*. Using the unique personal identification number of the cohort members, we traced residential histories in the CRS between 1971 and 2006. Each residential address contained a unique identification code composed of a municipality, road, and house number code. The dates the persons had moved to and from each address were noted. The addresses were then linked to a database of all official addresses and their geographical coordinates in Denmark. Geographical coordinates were obtained for 98% of all the residential addresses of the cohort members.

*Water supply and arsenic exposure*. The methods of obtaining arsenic concentrations in Danish drinking water for the cohort participants has been previously described in detail (Baastrup et al. 2008). In brief, arsenic concentrations were obtained from a database managed by the Geological Survey of Denmark and Greenland (Ministry of Climate Energy and Building 2014), which is the most important national source of information in Denmark on the conditions of groundwater, aquifers, and tap water sent to consumers (Thomsen et al. 2004). Different analytical methods were used for measuring arsenic in Danish drinking water throughout the investigated period relevant for the present study. However, the majority of the analyses were performed with inductively coupled plasma–mass spectrometry (ICP-MS) and ICP–atomic emission spectroscopy. We assumed a detection limit for these methods of 0.03–0.1 μg/L. The spatial locations of water utilities were determined by their geographical coordinates, also registered in that database. Average arsenic concentrations for each utility were calculated based on the 4,954 compulsory measurements taken at the outlet water pipe sending tap water to consumers in 2,487 water utilities from 1987 to 2004. This average was assumed to be representative of the arsenic concentrations throughout the study period of 1971–2006. In the geographical areas most densely populated with study participants, we mapped the geographical areas supplied by each water-supply unit, thus covering 76% of addresses, which enabled linkage of each address to the exact water-supply unit delivering drinking water to the household. For the remaining 24% of addresses, the “nearest water-supply unit approach” was applied. Volume-weighted arsenic concentrations were calculated for the areas receiving water from more than one utility. Using ArcMap, version 10.1 (ESRI), we linked the geocoded cohort addresses with water-supply areas or water utilities and their associated arsenic concentrations (Baastrup et al. 2008).

The time-weighted average (TWA) arsenic exposure (in micrograms per liter) was calculated as the arsenic concentration in drinking water multiplied by time lived at each address, summed for all addresses lived at during the study period, and divided by total observation time.

*Statistical methods*. The analyses were based on a Cox proportional hazards model with age as the underlying time scale ensuring that risk estimates were based on individuals at exactly the same age (Thiebaut and Benichou 2004). We used left truncation at age of recruitment so that people were considered at risk from enrollment into the cohort, and right censoring at the age of diabetes (event), death, emigration, disappearance, or end of follow-up on 31 December 2006, whichever came first, separately for the two definitions of diabetes. People diagnosed with diabetes before enrollment were excluded from the analyses.

Exposure, expressed as TWA arsenic in water since 1 January 1971, was entered into the statistical diabetes risk models as a time-dependent variable; thus recalculating exposure for noncensored persons at the time of each censor. The effects of arsenic exposure on diabetes was evaluated in several steps with adjustment for *a priori* defined confounders: *a*) adjusted only for age; and *b*) also including calendar year to account for time trends in diabetes incidence over follow-up) and full adjustment for the following recognized diabetes risk factors: sex, body mass index (BMI), waist circumference (in centimeters), smoking status, environmental tobacco smoke (ETS), leisure-time physical activity, alcohol intake, fruit intake, vegetable intake, saturated fat intake, educational level, and SES.

The risk factor ETS indicated living with a smoker and/or exposure to secondhand smoke at work for minimum of 4 hr/day. SES was based on municipality/district information on education, work-market affiliation, and income of the municipality/district that each cohort participant had lived in at the time of enrollment. Data on individual dietary intake of vegetables, fruit, and other foods were obtained from detailed self-administered, interviewer-checked, food frequency questionnaires. The dietary questions were designed to collect information about dietary habits; participants were asked how often on average they had consumed the different types of foods during the preceding 12 months. The frequency of consumption was categorized into 12 groups ranging from never to ≥ 8 times a day. A mean daily intake of foods (in grams per day) was calculated by multiplying the frequencies of intake by a sex-specific portion size using the software program FoodCalc, version 1.3 (Lauritsen 2004).

We formed four intervals for arsenic exposure using the 25th, 50th, and 75th percentiles for TWA arsenic since 1971 for all participants as the cut-off points and estimated the incidence rate ratios (IRRs) for the higher exposure ranges compared with the lowest exposure range. IRRs were also estimated as linear trends in arsenic concentrations.

Potential modifiers of an effect between arsenic exposure and diabetes included waist circumference, length of education, smoking status, and leisure-time physical activity (cycling, walking, and sports) as well as comorbid conditions (cardiovascular disease, including myocardial infarction and/or stroke at enrollment); effect modification was evaluated by introducing interaction terms into the adjusted model and using the Wald’s test. Because arsenic concentrations were generally higher for persons enrolled in the Aarhus area than for those in the Copenhagen area, we included separate analyses of risk in models stratified by enrollment area, knowing that results of such stratifications were limited because some participants had changed residences throughout the study period such that, overall, 11% and 14% lived outside the Copenhagen and Aarhus areas in 1971 and at the end of follow-up, respectively.

The results are expressed as IRRs with two-sided 95% confidence intervals (CIs) on the basis of the Wald test statistic for regression parameters in SAS (version 9.2; SAS Institute Inc.), whereas exposure–response curves with 95% confidence limits were visualized using a restricted cubic spline in R (library Survival and Design, version 2.13.1, R Project for Statistical Computing; http://www.r-project.org/) (Harrel 2001).

## Results

Among the 57,053 cohort members, 571 were excluded due to a cancer diagnosis before enrollment; 2 due to an uncertain date of cancer diagnosis; 1,191 due to self-reported diabetes before enrollment, a diabetes record in NDR before baseline, or having a diabetes diagnosis from NDR between baseline and 1 January 1995; 960 because their address history was not available in the CRS or their baseline address could not be geocoded, and 1,398 due to missing data in their potential confounders or arsenic-exposure information. The eligible 52,931 participants had lived in a total of 135,601 addresses and were followed up for diabetes for an average of 9.7 years. We identified 4,304 (8.1%) cases of diabetes in total (incidence rate 8.4/1,000 person-years). Of these, 1,269 cases that had been included in the NDR solely because of blood glucose measurements (without the results of those tests or diabetes confirmation in other registers) were excluded in a more strict definition of diabetes, resulting in 3,035 (5.8%) strict diabetes cases (incidence rate 5.9/1,000 person-years).

**Figure 1:**
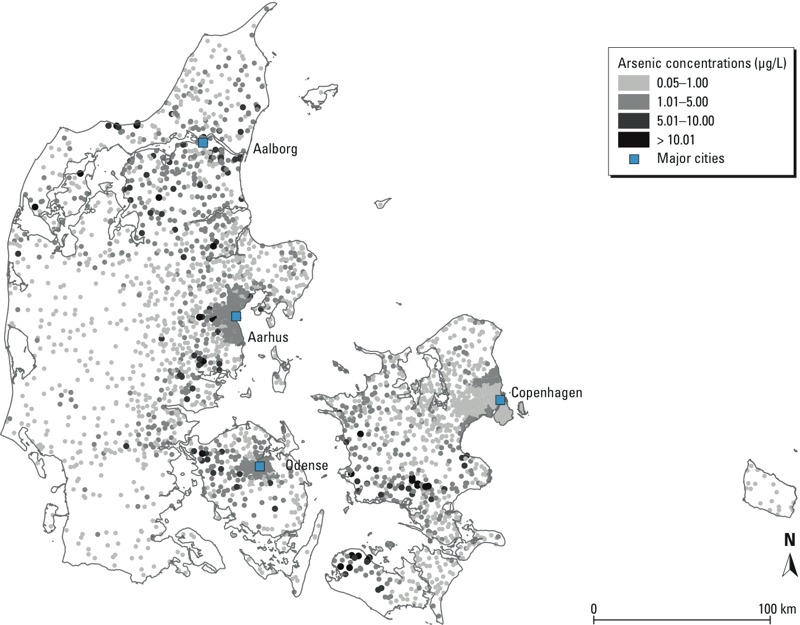
Arsenic concentrations for each utility in Denmark were calculated based on 4,954 compulsory measurements taken by 2,487 water utilities (1984–2004).

**Figure 2:**
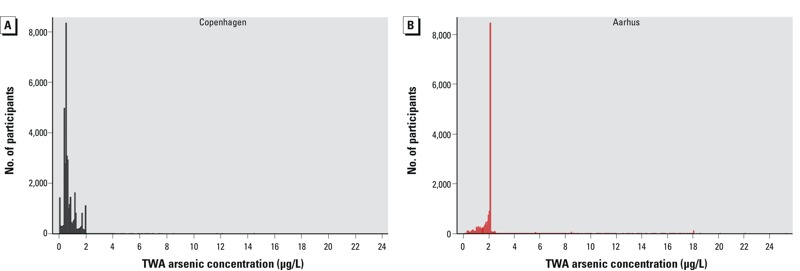
Distribution of TWA concentrations of arsenic from 1971 to the end of follow-up, stratified according to enrollment area of cohort participants. (*A*) Copenhagen (*n* = 39,698); mean (minimum–maximum) = 0.76 (0.05–11.0) μg/L; median (5th–95th percentile) = 0.58 (0.22–1.78) μg/L. (*B*) Aarhus (*n* = 16,233); mean (minimum–maximum) = 2.23 (0.09–25.3) μg/L; median (5th–95th percentile) = 2.11 (0.91–2.91) μg/L.

Diabetes cases were older at enrollment; had higher BMI and waist circumference, and higher alcohol and saturated fat intake; had lower education and SES; consumed fewer fruits and vegetables; and were more likely to be male, unemployed, current or previous smokers, exposed to ETS, and physically inactive; and had been exposed to similar arsenic concentrations compared with the whole cohort. Tap water was the major source of water intake, with a median intake of 1.6 L/day at enrollment (Table 1). The geographical variation of arsenic concentrations in Denmark is depicted in Figure 1. Water utilities in the Aarhus area distributed water with higher arsenic concentrations to consumers compared with those in the Copenhagen area. When considering the distribution of time-weighted arsenic concentrations since 1971 according to area of enrollment, concentrations among persons enrolled in Aarhus were higher, with a median of 2.11 μg/L compared with 0.58 μg/L in Copenhagen (Figure 2).

**Table 1:** Characteristics of the cohort by incident diabetes status at follow-up, for two definitions of diabetes based on the Danish NDR [*n* (%) or median (5th, 95th percentile)].

Characteristic	Total cohort	All diabetes^*a*^	Strict diabetes^*b*^
Population	52,931 (100)	4,304 (100)	3,035 (100)
Age (years)	56.1 (50.7, 64.2)	57.1 (50.9, 64.4)	57.0 (50.8, 64.5)
Males	25,102 (47.4)	2,438 (56.6)	1,807 (59.5)
BMI (kg/m^2^)	25.5 (20.4, 33.2)	28.5 (22.3, 38.0)	29.1 (22.8, 38.8)
Underweight (BMI < 18.5)	444 (0.8)	13 (0.3)	9 (0.3)
Normal weight (18.5 ≤ BMI < 25)	23,013 (43.5)	814 (18.9)	436 (14.4)
Overweight (25 ≤ BMI < 30)	22,040 (41.6)	1,880 (43.7)	1,324 (43.6)
Obese (BMI ≥ 30)	7,434 (14.0)	1,597 (37.1)	1,266 (41.7)
Waist circumference (cm)	88 (69, 110)	98 (75, 121)	100 (78, 122)
Length of education (years)
< 8	17,245 (32.6)	1,699 (39.5)	1,260 (41.5)
8–10	24,468 (46.2)	1,923 (44.7)	1,330 (43.8)
> 10	11,218 (21.2)	682 (15.9)	445 (14.7)
Occupational status
Employed	41,614 (78.6)	3,144 (73.0)	2,185 (72.0)
Unemployed/retired	11,317 (21.4)	1,160 (27.0)	850 (28.0)
SES^*c*^
Low	7,514 (14.2)	690 (16.0)	585 (19.3)
Low/medium	24,214 (45.8)	1,902 (44.2)	1,366 (45.0)
Medium/high	9,814 (18.5)	783 (18.2)	594 (19.6)
High	11,389 (21.5)	929 (21.6)	492 (16.1)
Smoking
Never	18,790 (35.5)	1,274 (29.6)	861 (28.4)
Previous	14,874 (28.1)	1,316 (30.6)	904 (29.8)
Current	19,267 (36.4)	1,714 (39.8)	1,270 (41.8)
ETS	33,809 (63.9)	2,944 (68.4)	2,126 (70.1)
Diet
Fruit intake (g/day)	145 (22.9, 472)	143 (19.7, 463)	138 (18.6, 454)
Vegetable intake (g/day)	158 (48.1, 351)	140 (41.8, 332)	136 (39.8, 324)
Saturated fat intake (g/day)	31.1 (15.8, 55.2)	31.4 (15.7, 56.4)	31.7 (16.3, 57.4)
Total intake of tap water (L/day)^*d*^	1.63 (0.70, 2.90)	1.60 (0.60, 2.80)	1.60 (0.56, 2.81)
Alcohol use	49,948 (94.4)	3,984 (92.6)	2,794 (92.1)
Cumulative alcohol use (g)^*e*^	14.1 (1.31, 65.3)	14.7 (1.00, 76.4)	15.0 (1.00, 80.1)
Activity
Physically active or play sports in leisure time	28,754 (54.3)	1,900 (44.1)	1,243 (41.0)
Physical activity (hr/week)^*f*^	2.0 (0.5, 7.0)	2.0 (0.5, 6.0)	2.0 (0.5, 6.0)
Arsenic at baseline (μg/L)	0.70 (0.05, 2.11)	0.70 (0.05, 2.11)	0.70 (0.43, 2.11)
^***a***^Includes hospital admissions for diabetes, diabetes medication, reimbursement for chiropody due to diabetes, or glucose blood tests. ^***b***^Excludes cases that were based solely on blood tests. ^***c***^Based on work-market affiliation, income, and education standards for the municipality each person lived in at enrollment. ^***d***^Total sum of tap water, coffee, tea, and fruit syrup/cordial diluted with tap water that was reported at enrollment. ^***e***^Based on all alcohol drinkers. ^***f***^Based on “physically active/play sports in leisure time” participants.			

**Figure 3:**
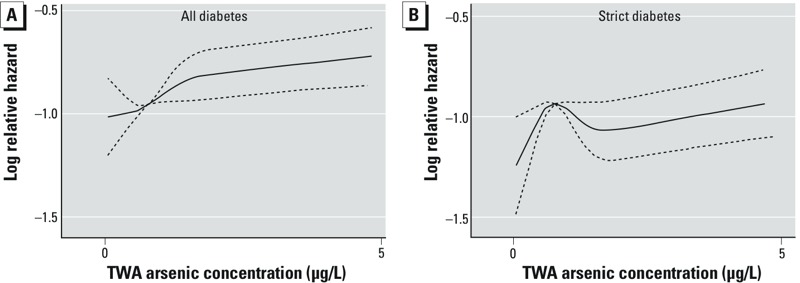
Spline functions (solid lines) between all diabetes (*A*) and strict diabetes (*B*) and average arsenic concentration at residences from 1971 on, based on fully adjusted models and cohort participants with exposure between the 1st and 99th percentiles; dashed lines indicate 95% CIs.

Overall, the adjusted IRR for all diabetes cases associated with each 1-μg/L increment increase in average arsenic levels was 1.03 (95% CI: 1.01, 1.06), whereas the adjusted IRR for strict diabetes was 1.02 (95% CI: 0.99, 1.05). There was no exposure dependence over the four arsenic exposure quartiles (Table 2), and we found no significant effect modifications (Table 3). Figure 3 shows increasing IRRs for all diabetes, with an increase in time-weighted arsenic exposure at the residential address since 1971, based on the fully adjusted models; for strict diabetes cases, this relationship showed an inverse U-shape in the lower exposure range and a slightly increasing IRR at the higher concentrations; CIs were wide.

**Table 2:** Association between arsenic exposure and diabetes among 52,931 DCH cohort participants for two definitions of diabetes incidence based on the NDR.

Diabetes definition and arsenic exposure (μg/L)	Cases (*n*)	IRR (95% CI)	
		Crude^*a*^^,^^*b*^	Adjusted model^*a*^^,^^*b*^^,^^*c*^
All diabetes			
< 0.57	1,049	1.00 (Referent)	1.00 (Referent)
0.57–0.74	1,021	1.09 (1.00, 1.19)	0.96 (0.87, 1.05)
0.74–1.82	1,017	1.06 (0.97, 1.15)	1.08 (0.99, 1.18)
> 1.82	1,217	1.20 (1.10, 1.30)	1.19 (1.09, 1.31)
Linear trend per μg/L	4,304	1.02 (1.01, 1.04)	1.03 (1.01, 1.06)
Strict diabetes
< 0.57	749	1.00 (Referent)	1.00 (Referent)
0.57–0.74	856	1.28 (1.16, 1.41)	1.03 (0.92, 1.14)
0.74–1.82	648	0.94 (0.84, 1.04)	1.00 (0.89, 1.11)
> 1.82	782	1.07 (0.97, 1.19)	0.99 (0.89, 1.11)
Linear trend per μg/L	3,035	1.00 (0.98, 1.03)	1.02 (0.99, 1.05)
^***a***^Adjusted for age by using it as the time scale in the Cox model. ^***b***^Because cohort members with missing value in any covariate were excluded, the number of persons was identical in the crude and the adjusted analyses. ^***c***^Adjusted for calendar year, sex, BMI (kg/m^2^), waist circumference (cm), smoking (indicator: never, former, current), ETS (indicator: yes/no), physical sports and activity in leisure time [indicator: yes/no, and intensity (hr/week)], alcohol consumption (indicator: yes/no, and g/day), fruit consumption (g/day), vegetable consumption (g/day), saturated fat consumption (g/day), educational level (indicator: < 8, 8–10, > 10 years), SES (indicator: low, medium low, medium high, and high).			

**Table 3:** Modifications of associations between TWA arsenic exposurea (per μg/L) and all diabetes cases (*n* = 4,303) among the 52,931 DCH cohort participants.

Potential effect modifier	Cases (*n*)	IRR (95% CI)^*b*^	*p*-Value^*c*^
Sex
Male	2,438	1.02 (0.99, 1.05)	0.11
Female	1,866	1.05 (1.02, 1.09)
Education (years)
< 8	1,699	1.05 (1.02, 1.08)	0.53
≥ 8	2,605	1.04 (1.01, 1.07)
Smoking status
Never	1,274	1.01 (0.96, 1.06)	0.62
Previous/current	3,030	1.02 (0.99, 1.05)
Physical activity
Yes	1,900	1.02 (1.01, 1.06)	0.98
No	2,404	1.03 (1.00, 1.06)
Waist circumference (cm)^*d*^
Low	1,007	1.02 (0.99, 1.05)	0.15
High	3,297	1.06 (0.99, 1.05)
Cardiovascular disease^*e*^
Yes	223	1.07 (0.97, 1.13)	0.69
No	4,081	1.03 (1.01, 1.05)
Enrollment clinic
Copenhagen	2,137	0.99 (0.90, 1.09)	0.43
Aarhus	898	1.03 (1.00, 1.06)
^***a***^Arsenic exposure was entered as a continuous variable in all models as the TWA concentration (μg/L) in tap water at residences from 1 January 1971 until censoring. ^***b***^The analysis was adjusted for age (underlying time scale), calendar year, sex (indicator: male, female), BMI (kg/m^2^), waist circumference (cm), smoking (indicator: never, former, present), ETS (indicator: yes/no), physical sports and activity in leisure time [indicator: yes/no and intensity (hr/week)], alcohol consumption (indicator: yes/no and g/day), fruit consumption (g/day), vegetable consumption (g/day), saturated fat consumption (g/day), educational level (indicator: < 8, 8–10, > 10 years), SES (indicator: low, low-medium, medium-high, high); however, with no adjustment for the modification variable. IRR expressed per μg/L of arsenic exposure. ^***c***^Test of the null hypothesis that the linear trends are identical, for Wald test for interaction. ^***d***^High waist circumference was defined as waist circumference > 102 cm in men and > 88 cm in women according to Lee et al. (2012). ^***e***^Including stroke and/or myocardial infarction at enrollment.			

## Discussion

We found that the risk of diabetes was positively associated with long-term exposure to low-level arsenic in drinking water.

This prospective cohort study related low-level arsenic exposure in tap water used for drinking, traced to each individual address, to the incidence of diabetes assessed objectively from a nationwide register, where information on a wide range of potential confounding factors was collected at enrollment without potential for recall bias. Previous studies of low-to-moderate arsenic exposure and diabetes risk used self-reports of diabetes and/or physician records of fasting blood glucose/75-g oral glucose tolerance tests (Coronado-González et al. 2007; James et al. 2013; Jovanovic et al. 2013; Kim et al. 2013; Li et al. 2013; Navas-Acien et al. 2008, 2009; Steinmaus et al. 2009; Wang et al. 2007; Zierold et al. 2004). In the present study, we relied on records of incident diabetes during follow-up in the NDR, not information such as self-reports or physician records of fasting blood glucose/75-g oral glucose tolerance tests as used in previous studies. The use of objective measures of diabetes based on a nationwide register, the NDR, is attractive because the entire population can be covered by uniform inclusion and objective criteria and the drop-out rate is zero (Carstensen et al. 2008). Also, all Danish citizens have free access to the health care system; thus, capture of diabetes within the entire population in the registry is assumed to be relatively free of SES bias that could limit study analyses in countries that do not have free health care access.

Among the limitations of the NDR is the lack of information regarding whether the registered diabetes was type 1 or type 2. However, type 2 diabetes generally constitutes 90–95% of all diabetes in this age group, and cohort participants who reported a diagnosis of diabetes before enrollment were excluded. Further limitations include that the date of inclusion in the NDR register was only a proxy for the diagnosis, which was likely actually made some time before inclusion in the NDR (Glümer et al. 2003). In addition, the NDR likely underestimated the actual diabetes burden because people without clinical diagnoses were not included.

Information on the level of blood glucose or other clinical measurements used at diagnosis is not available in the NDR. The “all diabetes” definition of incidence in the NDR has previously been validated by a study comparing register-identified patients with their general practitioners, and the sensitivity and predictive value of the criteria used in the NDR was found to be > 86% (Carstensen et al. 2008). However, the definition of incidence used in the NDR is based on four inclusion criteria, and three of these—hospital discharge diagnoses, chiropody, and diabetes medication records—reflect highly likely diabetes cases (“strict diabetes” cases), but the fourth—regarding blood glucose measurements—may lead to the inclusion of nondiabetic persons. No information is available on the results of the glucose tests or other records in the NDR for persons included solely on the basis of blood glucose measurements. Without this information, it is not possible to confirm diabetes among these persons, although the positive predictive value was 86% and 95%, respectively, for five measurements in 1 year and two measurements per year in 5-year periods in a validation exercise (Carstensen et al. 2008). Some of those people may not have had diabetes because there is an increasing awareness among physicians in Denmark regarding the detection of undiagnosed diabetes. Thus, it is common for elderly healthy people to have five blood glucose tests per year. To account for this, we included a second, strict definition of diabetes in which we excluded the 1,269 cases of diabetes that were based solely on blood glucose tests. The results of the analyses considering strict diabetes were slightly weaker than the results of those including all diabetes in this population, possibly questioning the causality of the relationship.

In this prospective cohort study, we found that diabetes risk was related to individual estimates of arsenic exposure in tap water used for drinking water by consumers based on geographical and registry linkage. The inclusion of the residential address history of the 53,931 eligible participants in as many as 135,601 individual homes, over a period of 35 years, added valuable strength to this study given that type 2 diabetes develops over many years (Genuth et al. 2003) and is often diagnosed some years after its actual onset (Glümer et al. 2003). One other very recent prospective case–cohort study that included 141 cases and 488 participants also related diabetes risk to arsenic in drinking water on the basis of lifetime reconstruction of exposure through structured interviews and geospatial modelling of groundwater inorganic arsenic concentrations (James et al. 2013). James et al. (2013) also reported an increased risk associated with individual exposure; although that study was smaller than the present study, the authors included the validation of estimated exposure using urinary inorganic arsenic species concentrations. The use of validation by James et al. (2013) is a clear strength when compared to the present study or other previous studies that used average arsenic in the area as a proxy of exposure, with no information on individual exposure histories (Islam et al. 2012; Jovanovic et al. 2013; Makris et al. 2012), which would imply some exposure misclassification. Another limitation of the exposure assessment used in the present study is that the residential histories of the cohort participants before 1971 were unknown, thus we could not assess the impact of early-life arsenic exposure. The lack of these older address histories suggest the possibility of some misclassification due to different migration patterns before 1971 for cases and noncases. In addition, we assumed that arsenic measurements taken after 1987 were representative of historical exposure, which would inherently be associated with some exposure misclassification.

In the present study, we included no information regarding exposure to organic arsenic via the diet (fish, shellfish, rice, and wine); however, such exposure is less harmful. Fish and shellfish are considered to be the most important sources of organic arsenic in the diet, and although fish and shellfish are included in the Danish diet, levels of consumption are low in a worldwide perspective when compared with central Asia or countries such as Japan. Further, we do not expect the typical Danish diet to include much of other high-arsenic foods such as seaweed. Addressing the potential effect of arsenic in seafood was not feasible because that adjustment would likely have led to other positive associations between seafood intake per se and diabetes risk, as Zhang et al. (2013) recently discussed in a comprehensive systematic review.

Finally, our estimation of arsenic exposure was based on the arsenic concentration in tap water at home as measured at the water utilities outlets providing drinking water to consumers, and although residential histories are accounted for, we cannot account for temporary migration and water sources at work in other regions in Denmark or while overseas in areas with either higher or lower levels of groundwater arsenic. Measurement of arsenic in nails, hair, or urine would provide more precise estimates of personal exposure but was not feasible in the present study. Measurement of arsenic in urine has previously been used in several studies, but most of those studies considered areas of high-level arsenic exposure. Among the studies considering moderate-level arsenic exposure, two reported significant positive associations between moderate arsenic exposure and diabetes risk (Coronado-González et al. 2007; Navas-Acien et al. 2008, 2009) and another reported a nonsignificant increased risk (Steinmaus et al. 2009).

Our analyses of risk were adjusted for dietary intake of vegetables, fruit, and other foods based on self-administered reports at enrollment; these dietary intakes would inevitably be affected by some degree of uncertainty and were only assessed at baseline. The participants’ diets—as well as lifestyle factors such as smoking—may have changed during the study. However, this misclassification would be nondifferential and unrelated to either the disease or arsenic exposure.

The importance of understanding the effects of low-to-moderate–level arsenic exposure in drinking water on diabetes has led to an increased interest in this association within the last decade. However, whereas evidence appears to be consistent at high levels of exposure, risk at low levels and possible thresholds have not been well addressed. The median arsenic exposure level at enrollment in the present study was 0.7 μg/L, which is well below the Danish guideline of 5 μg/L (Danish Ministry of the Environment 2007) and WHO guidelines of 10 μg/L (WHO 2006) and is comparable to the concentrations found in other northern European countries such as Finland (median, 0.14 μg/L) as well as in the United States (mean, 2 μg/L) (Agency for Toxic Substances and Disease Registry 2007). Our results indicate that exposure to low-level arsenic may play a small role in the diabetes epidemic and that risk could possibly increase by 2–3% per 1-μg/L increase in arsenic in drinking water.

## Conclusion

In a large prospective cohort study, we found a weak positive association between low-level arsenic concentrations in drinking water and the risk of diabetes. More work is needed to elucidate the role of low-level arsenic in the present diabetes epidemic.
